# Death wishes and death thoughts in paediatric palliative care: a survey of German healthcare professionals

**DOI:** 10.1186/s12904-025-01973-2

**Published:** 2025-12-12

**Authors:** Francesca Alt, Marie A. Neu, Hamideh Frühwein, Claudia Bozzaro, Jörg Faber, Norbert W. Paul

**Affiliations:** 1 University Medical Center of the Johannes Gutenberg-University Mainz, Childhood Cancer Center Mainz, Langenbeckstrasse 1, Mainz, 55131 Germany; 2 Institute for the History, Philosophy and Ethics of Medicine, University Medical Center of the Johannes Gutenberg-University Mainz, Am Pulverturm 13, Mainz, 55131 Germany; 3 Institute of Ethics, History and Theory of Medicine, University Medical Center of the University Münster, Von-Esmarch-Straße 62, Münster, 48149 Germany

**Keywords:** Paediatric palliative care, Death wishes, Death thoughts, Professional uncertainty, End of life communication, Ethical guidance

## Abstract

**Background:**

In paediatric palliative care (PPC), expressions related to death range from diffuse thoughts of dying “death thoughts” (DT) to explicit articulations of a desire to die “death wishes” (DW). These expressions pose significant ethical, clinical, and communicative challenges. Although often conflated in public discourse, DT and DW are conceptually distinct and have different implications for care, communication, and ethical reflection. This survey aimed to investigate how healthcare professionals (HCP) in Germany perceive, differentiate, and respond to these expressions in PPC practice.

**Methods:**

A nationwide online survey was conducted among multidisciplinary PPC professionals in Germany. The 43-item questionnaire included closed- and open-ended questions, addressing clinical experiences with death-related expressions in minors, professional responses, and institutional handling. Quantitative data were analysed descriptively. Qualitative free-text responses were analysed using a framework-guided hybrid thematic analysis.

**Results:**

A total of 120 HCP participated, including physicians (45%), nurses and social workers (13.3% each), psychologists (9.2%), and chaplains (7.5%). Experiences with DT were reported by 62 of 92 (67.4%) of HCP and DW by 62 of 93 (66.7%). Expressions were most observed in adolescents aged 15–18. DT were often reflective or symbolic, while DW tended to be more explicit and associated with suffering. Professional uncertainty was widespread: 49.3% reported feeling unsure about DT, and 57.5% about DW. Only 29.5% reported the presence of institutional guidelines. Still, 63.5% affirmed the clinical relevance of distinguishing DT from DW.

**Conclusions:**

These findings highlight a pressing need for conceptual clarity, ethical reflection, and institutional support in addressing death-related expressions in PPC. The distinction between DT and DW is clinically and ethically meaningful yet blurred in practice. Targeted training, evidence-based guidelines, and structured, interdisciplinary dialogue are essential to strengthen professionals’ confidence and competence in interpreting and managing these complex and ethically sensitive situations.

**Supplementary Information:**

The online version contains supplementary material available at 10.1186/s12904-025-01973-2.

## Background

The experience of dying is shaped not only by biomedical factors but also by profound existential, psychological, and relational dimensions. In adult palliative care, considerable attention has been given to patients who express a desire to die, a term that encompasses wishes for hastened death, acceptance of death, and ambivalence about the end of life [1–4]. Seminal studies by Chochinov et al. [5] established the multidimensional nature of these expressions, linking them to depression, demoralisation and a sense of meaninglessness. More recent studies have further emphasised the importance of differentiated clinical responses that consider the symbolic, communicative, or existential nature of such wishes [2, 6].

Adult patients usually have the cognitive and legal capacity to express such wishes and to participate in end-of-life decisions. This enables structured enquiry, guided by ethical principles such as autonomy, dignity and informed consent. In adult palliative care, tools such as the *Desire for Death Rating Scale (DDRS)* [5] and the German evidence-based guideline *Desire to Die in Palliative Care* [2] provide structured approaches to assessment and response. In contrast, paediatric expressions of death-related language remain poorly understood and far less frequently studied. Yet evidence suggests that children, particularly those with life-limiting conditions, also reflect on death and may express either transient or more deliberate death wishes (DW) [7–9]. While historically minimised or pathologised, such expressions are increasingly recognised by paediatric palliative and developmental scholars as developmentally appropriate and deserving of compassionate engagement, from early ethnographic accounts to more recent research emphasising children’s participation in decisions about dying [7–11].

Paediatric patients differ from adults in developmental capacity and in how existential distress is expressed, understood and responded to. Children often communicate distress through metaphor or behaviour rather than language [8, 9]. Moreover, the interpretive process is complicated by the involvement of caregivers and medical teams, who must interpret these expressions on behalf of the child. The ethical considerations are also unique: principles such as autonomy and informed consent are applied differently in paediatric care, requiring a more relational and context-sensitive approach [12–14].

Children’s expressions of distress frequently occur within a triadic care structure involving parents and professionals, further complicating assessment and response [13–15]. Parents may suppress or amplify their children’s expressions related to death, and professionals often find it difficult to distinguish between symbolic resistance, emotional exhaustion, and suicidal ideation [13, 15]. Even when paediatric palliative care (PPC) teams are highly experienced, research shows that many professionals still report uncertainty about how to interpret or respond to such expressions [6, 16].

Despite the growing recognition of the existential aspects of paediatric care, there is still a lack of empirical data on how professionals interpret and respond to expressions related to death. Clinical guidelines offer little structured direction, and institutional support for handling these ethically charged moments is inconsistent [2, 6]. As a result, professionals are often left to navigate ambiguous expressions without formal protocols, instead relying on subjective judgement, personal experience or team discussion. The lack of conceptual clarity, particularly between DW and death thoughts (DT), may lead to either an overreaction (e.g. an unnecessary psychiatric referral) or the neglect of a genuine existential or psychological need.

In Germany, PPC has evolved over the past two decades into a structured, multi-level system comprising hospital-based PPC units, paediatric hospices, and specialised outpatient PPC services (SAPPV). National policy initiatives and professional guidelines have helped establish PPC as a distinct field, yet coverage and training opportunities remain uneven across regions. According to current certification standards of the German Society for Palliative Medicine (DGP) and the statutory framework for specialised outpatient PPC, these services must operate as multidisciplinary teams. Each certified unit is required to include medical, nursing, psychosocial, and spiritual care professionals to ensure holistic, family-centred support.

In Germany, euthanasia (“*Tötung auf Verlangen”*, i.e. the intentional ending of a person’s life at their explicit request) remains prohibited under § 216 of the German Criminal Code. Assisted suicide, in contrast, is not criminally punishable following the 2020 decision of the Federal Constitutional Court, although no specific regulatory framework currently exists. For minors, both euthanasia and any form of assisted suicide are not permitted in clinical practice. These legal conditions shape professional understandings of death-related expressions and provide an important context for interpreting euthanasia-related inquiries within PPC.

This study aims to contribute to this emerging field by exploring how healthcare professionals (HCP) in Germany perceive, interpret and manage expressions of DT and DW in PPC. Using a combination of quantitative data and a framework-guided hybrid thematic analysis, the study seeks to clarify how these expressions are conceptualised in clinical practice, the ethical and communicative challenges they present, and how professionals navigate the blurred lines between developmental, existential and pathological forms of death-related communication.

## Methods

### Survey design and participants

This study employed a cross-sectional, mixed-methods design combining descriptive quantitative analysis with a framework-guided hybrid thematic analysis of open-text responses. An anonymous online survey was used to explore how HCP in PPC perceive and differentiate between DT and DW as expressed by minors. The survey was disseminated nationally through professional PPC networks, via the national network email list of the DGP, and PPC organisations across Germany.

The survey was created and administered using LimeSurvey (version 5.6; LimeSurvey GmbH, Hamburg, Germany). The platform allowed for adaptive item presentation and skip logic, ensuring that participants only viewed questions relevant to their professional background and previous responses. No identifying information, IP addresses, or metadata were stored, maintaining full anonymity throughout data collection.

Eligible participants were HCP and allied professionals working within specialist PPC settings, including paediatric hospices, hospital-based PPC units, and specialised outpatient PPC teams (SAPPV). The survey was open to all professional disciplines represented within these PPC settings, ensuring a multidisciplinary perspective. Therefore, participation was not limited to physicians or nurses but also included psychologists, social workers, grief counsellors, chaplains, physiotherapists, and art or music therapists, as well as other allied HCP involved in specialised PPC. A summary of participant characteristics, including professional roles, PPC experience, age, and religious affiliation, is presented in Table 2.

### Survey instrument

The survey was developed by a multidisciplinary research team with expertise in PPC, ethics, psychology, and qualitative research methods. The instrument comprised of 43 items, which were grouped into ten sections. The sections were designed to comprehensively cover relevant dimensions of the topic, including euthanasia, assisted suicide, DT, DW, communication practices, conceptual distinctions, professional uncertainties, and ethical recommendations. The questionnaire incorporated single- and multiple-choice items, ordinal-scaled responses, and open-text fields, thereby facilitating the collection of both quantitative and qualitative data. Participants were able to skip any item they did not wish to answer.

Items marked as “not reached” or “not viewed” in the dataset reflect LimeSurvey’s internal skip-logic mechanism, indicating that those questions were not displayed based on prior responses, rather than being actively skipped by participants.

To ensure conceptual clarity, participants were provided with standardised definitions of DT and DW drawn from established clinical and bioethical literature, offering a common interpretive framework ranging from general thoughts about mortality to explicit wishes to die.

The following definition of DT was provided: *“Death thoughts refer to thoughts about dying or being dead that arise consciously or unconsciously*,* often in the context of illness. These thoughts are not necessarily an expression of a specific desire to die. They may indicate a general sense of no longer wanting to live in such a condition*,* or reflect philosophical*,* spiritual or existential contemplation about the nature of death.”*

Respondents were informed that, for the purposes of the survey, a DW was defined as *“the desire not to continue living. It is a form of wishing for a shortened lifespan*,* often arising from the experience of intense suffering or a sense of futility. Suicidality may or may not be implied.”*

The instrument was piloted with five professionals from different disciplines, and feedback led to minor refinements in wording and structure. The survey design is outlined in Table 1.

**Table 1:** Overview of survey design

Component	Description
Study design	Cross-sectional, mixed-methods online survey
Target population	HCP in PPC
Inclusion criteria	Professional experience providing care for children or adolescents with life-limiting or life-threatening conditions within specialised PPC settings (paediatric hospices, hospital-based PPC professionals, or specialised outpatient PPC teams, SAPPV)
Recruitment channels	Mailing list of national network: “Deutsche Gesellschaft für Palliativmedizin”, “DGP” (German Association for Palliative Medicine); institutional mailing lists
Data collection method	Anonymous online questionnaire (LimeSurvey platform)
Number of items	43
Response formats	Single- and multiple-choice, ordinal-scaled items, open-text fields
Survey structure	Ten sections: demographics; euthanasia inquiries; assisted suicide; DT; DW; communication; conceptual distinctions; uncertainties; ethical/institutional needs; recommendations
Definitions provided	Standardised definitions of DT and DW based on clinical and bioethical literature
Data analysis	Quantitative data analysis: descriptive statistics; qualitative data analysis: framework-guided hybrid thematic analysis.

*Abbreviations*: *DT* Death thoughts, *DW* Death wishes

### Quantitative data analysis

Quantitative data were analysed descriptively using frequency distributions and percentages. The objective of these analyses was to characterise the prevalence and patterns of death-related expressions and to explore institutional and professional responses across roles and settings. Percentages refer to valid responses per item, and missing data were excluded from the denominator. In the dataset export, items marked as “*not reached or viewed”* refer to questions hidden by the survey’s skip logic rather than to participants’ active non-response.

### Qualitative data analysis

Qualitative free-text responses were analysed using a framework-guided hybrid thematic approach combining deductive organisation of the dataset with inductive, reflexive thematic development. This approach is consistent with published descriptions of hybrid deductive–inductive thematic analysis in applied health research [17, 18] and with Braun and Clarke’s principles of reflexive thematic analysis [19]. Because the free-text material consisted of short, heterogeneous comments linked to specific survey domains (euthanasia-related inquiries, DT, DW, communication practices, professional uncertainties, institutional needs), the predefined structure of the questionnaire served as the deductive organising framework aligned with the conceptual areas that informed survey development. Inductive coding within each domain then enabled the development of data-driven patterns of meaning across the dataset.

Analysis proceeded in several stages. First, the primary coders (FA, MN) familiarised themselves with all free-text responses through repeated reading, noting early observations in line with recommendations for the familiarisation phase of thematic analysis [18, 19]. Second, responses were deductively sorted into the relevant questionnaire domains. This step facilitated systematic data management and maintained analytic alignment with the conceptual structure of the survey [17]. Third, within each domain, inductive coding was undertaken without the use of a predefined codebook. Codes were generated directly from participants’ language (e.g., “existential fear”, “metaphorical expression”, “uncertainty about intention”), consistent with reflexive thematic analysis [19]. Coding was iterative: FA and MN compared coding lists, discussed discrepancies, and refined code definitions through repeated engagement with the data [18]. Finally, conceptually related codes were grouped across domains into broader, cross-cutting themes. This stage involved recursive movement between codes, data extracts, and candidate themes, reflecting a reflexive and interpretive analytic process rather than a mechanical coding-tree approach. The resulting themes integrate both anticipated (deductively organised) and emergent (inductively developed) aspects of how HCP interpret and respond to DT and DW in PPC. Given the structurally brief nature of free-text responses, analysis focused on semantic, low-inference patterns that reflected professionals’ perspectives, while still allowing for interpretive synthesis across domains. Verbatim quotations are used in the Results to illustrate these themes.

### Researcher reflexivity

The analysis team comprised professionals from diverse disciplinary backgrounds: FA (physician, paediatric oncologist, PPC specialist and clinical ethicist), MN (physician and researcher), HF (public health researcher), NWP (philosopher, researcher and senior clinical ethicist), and JF (paediatric oncologist and researcher). We acknowledge that our clinical, ethical, and philosophical perspectives inevitably influenced the interpretation of participants’ statements. Reflexive discussions were held throughout coding and theme refinement to enhance transparency and to consider how our respective positionalities shaped the analytic focus.

Reporting follows the Consolidated Criteria for Reporting Qualitative Research (COREQ) to ensure transparency and completeness (Supplementary Material, Table 2).

### Ethical considerations

Participation was entirely voluntary and anonymous, with no identifiable data collected. All respondents were informed about the study’s purpose and data handling procedures prior to participation.

## Results

The results comprise two complementary components in line with the mixed-methods design: (1) descriptive quantitative findings and (2) qualitative thematic findings derived from free-text survey responses. The quantitative section summarises the frequency and distribution of HCP-reported experiences, while the qualitative analysis explores how professionals interpreted and made sense of children’s death-related expressions across survey domains.

Percentages refer to valid responses per item. Items noted as *“not viewed”* indicate that the question was hidden by the survey’s internal skip logic and therefore not displayed to the participant. Verbatim quotations from free-text responses are presented to illustrate HCP perspectives and experiences. In several quotations, participants use the first-person plural (“we”) to describe team-based or institutional practice, consistent with the phrasing of the survey questions.

### Demographics and professional background

Of the 120 participants, *n* = 111 (92.5%) completed the demographic and background section. Completion of these items was voluntary, and therefore not all participants provided demographic data. The percentages below refer to this subgroup of valid responses (*n* = 111), while missing responses are reported separately for each item.

Participants represented a range of professional backgrounds. The largest group identified as physicians (*n* = 50; 45.0%), followed by nurses (*n* = 16; 13.3%) and social workers (*n* = 16; 13.3%). Additional roles included psychologists (*n* = 11; 9.2%), chaplains or bereavement counsellors (*n* = 9; 7.5%), and physiotherapists (*n* = 4; 3.3%). One participant each (0.8%) reported working in art therapy and in a volunteer capacity. A further 10 (8.3%) selected “other,” describing roles in hospice care, education, or team coordination. Nine participants (7.5%) did not reach or respond to this item.

Among those who responded (*n* = 111), 38 participants (34.2%) had more than 10 years of experience in PPC, 28 (25.2%) had 5–10 years, and 33 (29.7%) had less than 5 years. Ten respondents (9.0%) selected “other.” Two (1.7%) skipped the item, and nine (7.5%) did not reach it. Of the 111 respondents, 85 (76.6%) identified as female, and 24 (21.6%) as male. None identified as diverse. Two participants (1.7%) skipped this item, and nine (7.5%) did not reach it.

Participants were most aged 40–49 years (*n* = 34; 30.6%) and 50–59 years (*n* = 32; 28.8%). Other age groups included 30–39 years (*n* = 21; 18.9%), 60–69 years (*n* = 12; 10.8%), and under 30 (*n* = 10; 9.0%). No participants were under 20 years. Two respondents (1.7%) skipped the item, and nine (7.5%) did not view it.

A total of 38 participants (34.2%) identified as Protestant, 31 (27.9%) as having no affiliation, and 30 (27.0%) as Roman Catholic. One participant identified as Muslim (*n* = 1; 0.9%) and nine (*n* = 9; 8.1%) selected “other.” Two respondents (1.7%) skipped the item, and nine (*n* = 9; 7.5%) did not reach or view the question. A summary of participants’ demographic and professional characteristics is presented in Table 2.

**Table 2:** Demographic and professional characteristics of participants (*n* = 120)

Characteristic	Category	*n* (%)
Completion of demographic section	Completed	111 (92.5%)
	Not completed	9 (7.5%)
Professional background (*all participants employed within PPC settings)*	Physician	50 (45.0%)
	Nurse	16 (13.3%)
	Social worker	16 (13.3%)
	Psychologist	11 (9.2%)
	Chaplain / bereavement counsellor	9 (7.5%)
	Physiotherapist	4 (3.3%)
	Art therapist	1 (0.8%)
	Volunteer	1 (0.8%)
	Other (e.g. hospice, education, coordination)	10 (8.3%)
Years of experience in PPC	< 5 years	33 (29.7%)
	5–10 years	28 (25.2%)
	> 10 years	38 (34.2%)
	Other	10 (9.0%)
Gender	Female	85 (76.6%)
	Male	24 (21.6%)
	Diverse / other	0 (0.0%)
Age group (years)	< 30	10 (9.0%)
	30–39	21 (18.9%)
	40–49	34 (30.6%)
	50–59	32 (28.8%)
	60–69	12 (10.8%)
Religious affiliation	Protestant	38 (34.2%)
	Roman Catholic	30 (27.0%)
	None	31 (27.9%)
	Muslim	1 (0.9%)
	Other	9 (8.1%)

Percentages refer to valid responses per item (*n* = 111 for demographic questions)

### Experiences with euthanasia-related inquiries

Of the 120 participants, 106 (88.3%) responded to the question about whether they had ever been approached with questions regarding euthanasia in the context of PPC. Of these, 50 (47.2% of valid responses; 41.7% of total sample) answered affirmatively.

Among those who reported such encounters (*n* = 50), the most common frequency was “one to three times” (*n* = 26; 52.0%), followed by “four to six times” (*n* = 12; 24.0%), “more than ten times” (*n* = 9; 18.0%), and “seven to nine times” (*n* = 3; 6.0%). These follow-up responses were displayed only to participants who had selected “yes”; 70 (58.3%) did not view these items due to skip logic.

Regarding the source of these statements, 50 respondents provided information. The majority reported that euthanasia-related questions came from parents (*n* = 34; 68.0%), followed by patients themselves (*n* = 24; 48.0%), siblings (*n* = 9; 18.0%), grandparents (*n* = 7; 14.0%), and other family members or caregivers (*n* = 11; 22.0%). As with the previous item, 70 (58.3% of total sample) did not reach or view this follow-up item due to survey logic.

### Encounters with assisted suicide requests

Direct requests for assisted suicide from paediatric patients, as reported by HCP, were reported by a small minority of respondents. Only 5 (4.9%) of 103 respondents indicated that they had experienced such requests. All reported cases involved adolescents, with three patients aged 15–18 years (*n* = 3; 2.5%) and one aged 12–14 years (*n* = 1; 0.8%). No requests were reported from children under the age of 12. The underlying diagnoses in these cases included oncological conditions (*n* = 3; 60%) and genetic disorders (*n* = 2; 40%).

One professional provided a direct quotation illustrating such an encounter:

*“If you could give me something*,* I would legally end my life.”* (Quote AS-01).

### Healthcare professionals’ experiences with children’s death-related expressions

#### Death thoughts (DT)

A total of 92 participants (76.7%) responded to the question about whether they had encountered DT expressed by paediatric patients. Of these, 62 (67.4%) reported such experiences, while 30 (32.6%) had not. One participant (0.8%) skipped the question, and 27 (22.5%) did not view the item due to skip logic.

Among those who had encountered DT (*n* = 62), the majority (*n* = 38; 61.3%) indicated they had observed such expressions “one to three times”. Further responses included “four to six times” (*n* = 10; 16.1%), “seven to nine times” (*n* = 3; 4.8%), and “more than ten times” (*n* = 11; 17.7%).

Regarding the age of patients expressing DT (multiple responses allowed), most were 15–18 years (*n* = 38; 61.3%), followed by 12–14 years (*n* = 31; 50.0%) and 9–11 years (*n* = 21; 33.9%). DT were less frequently observed in younger children: 6–8 years (*n* = 11; 17.7%) and under five (*n* = 6; 9.7%).

Verbatim statements provided by 35 participants (29.2%) included symbolic imagery, references to fear, and indications such as giving away personal belongings, for example:

*“When I die*,* I want to be a star in the sky.”* (Quote DT-02).

*“I’m afraid to fall asleep because I may never wake up.”* (Quote DT-03).

*“I’m giving my things away.”* (Quote DT-01).

#### Death wishes (DW)

HCP experiences of DW expressions by paediatric patients were explored through a set of structured and open-ended questions.

A total of 93 participants (77.5%) responded to the question regarding whether they had encountered DW expressed by their patients. Of these, 62 (66.7% of valid responses; 51.7% of the total sample) reported having encountered such expressions, while 30 (32.3%) had not. One participant (0.8%) skipped the question, and 26 (21.7%) did not reach or view the item due to skip logic.

Among those who had encountered DW (*n* = 62), the majority (*n* = 38; 61.3%) had observed such expressions “one to three times”. Additional responses indicated “four to six times” (*n* = 10; 16.1%), “seven to nine times” (*n* = 3; 4.8%), and “more than ten times” (*n* = 11; 17.7%).

Participants also indicated the age groups in which DW had been expressed. Of those who answered this item (*n* = 38; 61.3%) associated DW with patients aged 15–18 years, 31 (50.0%) with those aged 12–14 years, 21 (33.9%) with children aged 9–11 years, 11 (17.7%) with children aged 6–8 years, and 6 (9.7%) with children under the age of five.

A total of 35 (29.2%) participants responded to the open-ended item on DW.

Of these, 17 provided direct quotations, such as:

*“I don’t want to live anymore.”* (Quote DW-01).

*“Can you do something to end this?”* (Quote DW-03).

*“I wish I wouldn’t wake up tomorrow.”* (Quote DW-03).

#### Communication practices of HCP in response to children’s DT or DW

Professionals were asked whether they actively addressed the topic of death and dying with paediatric patients, and whether institutional guidance such as a standard operating procedure (SOP) was available.

Of the 120 participants, 78 (65.0%) responded to the question concerning active communication practices with patients. Among them, 52 (66.7%) indicated that they do address these topics, while 26 (33.3%) stated that they do not. Forty-two (35.0%) did not reach or view the item due to skip logic.

The same number of participants (*n* = 78; 65.0%) responded to the question on institutional guidance. Of these, 23 (29.5%) reported that their team follows a formal SOP or internal guideline, while 54 (69.2%) reported that no such guidance is available. One participant (1.3%) skipped the question, and 42 (35.0%) did not view the item due to skip logic.

In the open-ended responses, professionals described age-appropriate and legally informed approaches to communication, for example:

*“We respond to expressed thoughts of death in an age-appropriate manner.”* (Quote F5F-01).

*“In the case of an explicitly expressed wish to die*,* we refer to legal limitations.”* (Quote F5F-02).

#### Perceived conceptual distinction between DT and DW (HCP perspectives)

This section explored whether HCP in PPC perceive a meaningful clinical distinction between DT and DW.

Of the 120 participants, 96 (80.0%) responded to the corresponding item. Among them, 61 (63.5%) affirmed the clinical relevance of differentiating between the two concepts, 17 (17.7%) saw no clinical benefit in doing so, and 18 (18.8%) were unsure. 24 (20.0%) did not reach or view the item.

#### Professional uncertainties in interpreting DT and DW

This section of the survey assessed the extent to which HCP in PPC experience uncertainty when interpreting and responding to thoughts and wishes relating to death specifically DT and DW.

Of the 120 participants, 73 (60.8%) participants responded to the question about uncertainty regarding DT. Among them, 36 (49.3%) reported experiencing uncertainty, 19 (26.0%) did not, and 18 (24.7%) were unsure. Forty-seven (39.2%) did not view the item due to skip logic.

The same number of participants (*n* = 73) responded to the corresponding item on DW, with 42 (57.5%) reporting uncertainty, 20 (27.4%) being unsure, and 11 (15.1%) not experiencing uncertainty. Forty-seven (39.2%) did not view the item due to skip logic.

### Institutional, ethical, and research needs identified by professionals

This section of the survey examined whether respondents considered further research and institutional support necessary for addressing death-related expression in PPC.

Of the 120 participants, 70 (58.3%) responded to the corresponding item. Among these, 58 (82.9%) affirmed the need for additional research and institutional guidance, 11 (15.7%) reported being unsure, and only one (1.4%) answered “no”. Fifty participants (41.7%) did not reach or respond to the item.

Among those who endorsed the need for further action (*n* = 58), 51 (87.9%) also provided concrete recommendations.

### Qualitative findings: cross-cutting themes from free-text responses

Analysis of the free-text responses across the survey identified three interrelated themes that describe how HCP interpret, differentiate, and respond to children’s expressions of DT and DW in PPC. These themes cut across all survey domains, including DT, DW, communication practices, conceptual distinctions, professional uncertainties, and institutional needs.

#### Theme 1. Interpreting children’s death-related expressions: developmental, symbolic, and existential meanings

Across disciplines, professionals described a wide range of children’s death-related expressions, from metaphorical or symbolic statements to explicit articulations. In their free-text responses, respondents recalled children using highly symbolic or imaginative language, such as asking, *“When am I allowed to go over the rainbow? And can you help me?”* (DT-148) or employing transformative imagery to cope with dying: *“When the time comes*,* I will just imagine turning into a sea turtle and gliding into an underwater world.”* (DT-382). Other recollected expressions reflected practical or ritual curiosity, for example: *“I want there to be spaghetti bolognese at my funeral.”* (DT-418), or existential questions including *“How does someone die? What does dying feel like? Will I be in pain?”* (DT-361). Professionals often interpreted these DT as part of children’s natural coping processes, representing fear, curiosity, or an attempt to make sense of illness.

In contrast, professionals remembered DW as more explicit, intentional, or affectively charged, often expressing emotional exhaustion, suffering, or a wish for relief. Examples included recollections of adolescents stating: *“I don’t want to live anymore.”* (DW-157), *“I cannot bear this any longer; I want to die.”* (DW-157), or *“I wish I would not wake up tomorrow morning.”* (DW-355). Some recollections reflected a more direct wish for hastening death, such as: *“Please give me something so that I can die.”* (DW-211) or *“I would like to die today if possible.”* (DW-220).

Several professionals highlighted the interpretive ambiguity of certain expressions. Statements such as *“I just want all of this to be over and to fall asleep peacefully.”* (DT/DW-172) were described as difficult to classify without contextual understanding. Others, such as *“Heaven must be nicer than here; I wish I were already there.”* (DW-205) or *“It will be easier for everyone if I die.”* (DW-361) were seen as spanning existential reflection, spiritual meaning-making, and relational despair. These variations illustrate how meaning is shaped by developmental level, illness stage, and family context.

Professionals emphasised that younger children more commonly used symbolic or fantastical language (e.g., *“I will get wings soon.”* (DT-430)), whereas adolescents were more often recalled as expressing deliberative or goal-directed DW, such as wanting to *“fall asleep and not wake up again”* (DW-268) or giving away belongings (DW-361). These recollections demonstrate the contextual and multi-layered nature of interpreting DT and DW in PPC.

Many professionals pointed to developmental factors as important for distinguishing between DT and DW. Younger children were described as more likely to express fears or fantasies, whereas adolescents were perceived as more capable of articulating deliberately phrased DW. Professionals from different disciplines emphasised different aspects of these expressions, such as psychological risk, spiritual distress, and family dynamics. This highlights the contextual and multi-layered nature of interpretation in PPC.

#### Theme 2. Professional uncertainty and the emotional-ethical burden of interpretation

A prominent theme across the open-text responses was the uncertainty and emotional burden professionals experienced when interpreting children’s death-related expressions. Respondents described struggling to discern whether a recalled statement reflected existential reflection, psychosocial distress, symbolic communication, or an explicit wish to die. This ambiguity was particularly evident in statements such as: *“I just want all of this to be over and to fall asleep peacefully.”* (DT/DW-172) or *“I cannot live like this anymore*,* can’t you do something?”* (DW-298), which professionals considered challenging to interpret without additional context. This interpretive tension is captured in questions such as: *“How serious is the patient – do they really want to die*,* are they seeking attention?”* (TU-01).

HCP across roles reported feeling insufficiently prepared to respond to explicit DW. Several expressed a fear of either overreacting (e.g., pathologising existential statements) or underestimating the situation (e.g., missing indicators of acute psychological risk):

*“I wouldn’t know how to appropriately handle explicit death wishes.”* (TU-02).

For some respondents, the recollection of adolescents expressing ideas such as wanting to *“fall asleep and not wake up again”* (DW-268) or asking to be *“let die”* (DW-349) created moral and emotional burden. Professionals described feeling responsible for interpreting such statements in light of developmental capacity, family dynamics, and the clinical trajectory. Many noted the absence of clear institutional frameworks or training to guide these decisions, contributing to feelings of uncertainty and emotional strain.

Interpretive uncertainty was often described as exacerbated by relational and institutional factors. Respondents noted that paediatric care involves triadic communication (child-parent-clinician), making interpretation more complex and increasing the emotional demands placed on professionals.

#### Theme 3. Structural gaps and the need for institutional and ethical support

Across survey domains, respondents consistently highlighted the absence of institutional structures, training, and formal guidance for addressing DT and DW in PPC. Only a minority reported access to SOP or internal guidelines, and many noted that their teams lacked shared frameworks for responding to these complex expressions. This lack of institutional support was perceived as contributing to considerable variability in practice and amplifying professionals’ uncertainty.

Free-text comments frequently referred to the need for clearer and more comprehensive organisational support. Several professionals described the absence of age-appropriate communication tools, limited opportunities for interdisciplinary ethics consultation, and a lack of defined escalation pathways for psychological or psychiatric assessment. Others pointed to the scarcity of structured team discussions or reflective spaces in which challenging cases could be examined collectively. Standardised internal guidance, such as SOP, or decision-support tools, was viewed as particularly important for creating consistency and reducing the reliance on individual judgement. As one professional summarised:*“We need more support to know how to respond appropriately - not just medically*,* but ethically and emotionally.”* (AB-01).

In addition to procedural clarity, respondents emphasised the importance of conceptual grounding. Some noted that without a robust, evidence-based understanding of how DT and DW differ across developmental stages, clinicians may struggle to interpret the significance of such statements. Others remarked that institutional guidance provides emotional reassurance and ethical containment, offering shared language and structured decision-making processes to support professionals in navigating sensitive, high-stakes conversations.

Finally, many participants underscored the need for further research to strengthen clinical and ethical practice in this area. Suggestions included investigating the causes and meanings of DW in children, integrating the perspectives of bereaved families, and developing age-appropriate tools for assessing existential distress. Several respondents also called for enhanced training programmes, reflective practice sessions, and structured interprofessional education to support professional competence, confidence, and emotional resilience when working with death-related expressions in PPC.

## Discussion

### Overview of main findings

This mixed-methods survey provides a comprehensive overview of how HCP in Germany encounter and interpret children’s expressions of DT and DW within PPC. As this study draws exclusively on professionals’ recollections and interpretations rather than direct patient accounts, all findings reflect HCP perceptions of children’s expressions within their clinical and institutional contexts. The findings reveal three overarching patterns: professionals differentiate DT from DW but often struggle with diagnostic and ethical ambiguity; institutional and educational frameworks for responding to these expressions remain limited; and participants express a strong desire for structured guidance, interdisciplinary support, and ethical reflection. Together, these results illustrate a tension between individual professional sensitivity and systemic gaps in training and policy.

Expressions of DT and DW by paediatric palliative patients are therefore both ethically charged and clinically complex. The following discussion unpacks these interrelated dimensions - clinical, institutional, and ethical - to illuminate key challenges and implications for PPC practice.

### Clinical interpretation and response

Many HCP reported encountering expressions related to death in their routine clinical practice. These expressions occurred across the age spectrum and were most frequently recollected among adolescents, consistent with known PPC population characteristics and symptom trajectories [14]. In line with previous research in adult palliative care, such as the Desire for Death Rating Scale [5] and the German evidence-based guideline “Desire to Die in Palliative Care” [6], our findings similarly reveal the need for structured interpretive and communicative frameworks. However, unlike adult care, PPC practice lacks validated instruments and ethical guidance adapted to children’s developmental stages. These adult frameworks provide structured ethical and communicative approaches but cannot be directly applied to paediatrics, where developmental and relational dynamics require distinct interpretive models.

In this study, professionals described difficulty in distinguishing DT, which is often perceived as existential and developmentally appropriate reflection, from DW. The latter was interpreted as more explicit and possibly indicative of suffering or acute psychological distress. These diagnostic ambiguities mirror findings in adult palliative care, where the desire to hasten death may coexist with an ongoing will to live, creating a complex interpretive landscape for clinicians [3, 5]. In paediatrics, interpretation is further complicated by the use of proxy communication through parents, as well as the absence of validated tools to assess existential distress in children. This further underlines the need for structured interpretive models and clinical communication tools to support practice in this area [6].

### Institutional and structural factors

Although most respondents had encountered death-related expressions, only a minority reported having access to formal institutional guidance; SOP or internal policies for managing such situations were described as rare. This structural gap appears to contribute to clinical uncertainty and inconsistency, echoing previous studies showing that PPC training frequently provides limited preparation for existential or developmentally sensitive communication [2, 9, 12]. Respondents noted that teams often rely on individual judgement or informal discussion rather than shared frameworks. This variability may lead to divergent responses to similar situations and reflects a broader imbalance in PPC training, which prioritises somatic symptom management over existential and psychosocial aspects of care. These findings suggest a need for systematic integration of communication and ethics training within PPC education.

### Ethical and conceptual ambiguities

Even in cases where professionals reported direct requests for assisted suicide, the quotations provided often remained open to interpretation. Respondents emphasised that children’s statements often blend metaphor, suffering, emotional overwhelm, or relational tension, making it difficult to infer intention. Several professionals described DW not as literal wishes for death but as symbolic expressions of exhaustion, withdrawal, or a desire for relief- echoing findings from caregiver studies on the need for interpretive support in end-of-life communication [15]. Ethical uncertainty therefore emerged as a central challenge. Although most professionals agreed that distinguishing between DT and DW is clinically meaningful, many reported difficulty applying this distinction consistently in practice. Some voiced concern about underreacting to expressions that may conceal suicidal ideation, while others feared overreacting and pathologising developmentally normative or existential statements.

This ethical tension, between respecting children’s inner lives and recognising their developmental and cognitive limitations, is exacerbated by the lack of a shared ethical frameworks and absence of institutional clarity. Respondents described discomfort within teams when managing such expressions. The absence of shared criteria for mental health referral, spiritual support, or care-plan modification was perceived as a barrier to ethically grounded responses. Without common frameworks, team members often relied on individual judgement or intuition, which increased emotional pressure and uncertainty.

Encouragingly, many participants underscored the need for further education and empirical research. They emphasised that communication about DT and DW requires clinical sensitivity, ethical competence, and interpretive skill, capacities that are not yet systematically supported across PPC environments. This call for improved ethical, developmental, and communicative guidance aligns with international priorities in PPC research.

### Practice and policy recommendations

The findings of this study point to several concrete recommendations for improving the clinical and ethical response to death-related expressions in PPC. The development of evidence-based practice guidelines is essential. Such protocols should offer clear distinctions between symbolic, existential, and potentially pathological expressions and provide structured support for documentation, triage, communication with families, and ethical deliberation in complex or ambiguous situations.

Targeted training and simulation-based education should be integrated into PPC curricula. Educational initiatives ought to focus specifically on communication around DT and DW and include simulation exercises as well as reflective case discussions. Particular attention should be paid to the needs of early-career clinicians, who reported lower levels of confidence in managing these expressions.

Such efforts could be informed by emerging international consensus-building approaches to end-of-life communication with young populations [13]. Regularly facilitated discussions involving chaplaincy, psychiatry, social work, and ethics consultation can help clarify responsibilities, promote shared understanding within teams, and reduce individual moral burden.

There is also a pressing need for the development and validation of structured, age-appropriate instruments to assess existential distress in children. The current lack of reliable tools impairs clinicians’ ability to distinguish between developmentally normative reflections on death and expressions indicating psychosocial or psychiatric risk. Empirically grounded assessment instruments would enable more nuanced and effective interventions and facilitate the generation of comparative data for future research.

### Limitations

This study has several limitations that should be acknowledged. First, participation was voluntary and recruitment occurred mainly through established PPC networks, which may have led to a response bias towards professionals with a particular interest or experience in ethical and communicative aspects of care. Second, the survey was open to a range of professional disciplines within specialised PPC settings only - paediatric hospices, hospital-based PPC units, and SAPPV teams- and therefore does not capture perspectives from non-specialist paediatric or acute-care environments. Third, the qualitative data consisted of free-text comments, which provided valuable insights but limited depth compared with interview-based designs. Fourth, the analysis relies entirely on professionals’ recollections of children’s expressions, which may differ from the children’s intended meanings; however, this approach is appropriate and methodologically transparent given the survey design. Fifth, although standardised definitions of DT and DW were provided to ensure conceptual clarity, no explicit definition of euthanasia was included; and interpretations of that term may have varied among respondents. Finally, the cross-sectional design captures professionals’ perceptions at a single point in time and cannot assess changes over time or causal relationships. Despite these limitations, the study offers an important overview of multidisciplinary experiences within PPC and identifies areas in which conceptual, communicative, and institutional guidance remains underdeveloped.

## Conclusion

In PPC, DT and DW represent distinct yet interrelated phenomena that necessitate responses which are both ethically sensitive and clinically robust. The findings of this study indicate that HCP working within specialised PPC settings frequently encounter such expressions and perceive them as ethically and emotionally significant elements of paediatric care. These insights reflect professionals’ interpretations within their clinical and institutional contexts, rather than direct accounts from patients’ inner experiences.

Despite their reported prevalence, the majority of professionals in this study described a lack of institutional protocols, structured training, and shared conceptual understanding to address these expressions effectively. Such gaps increase the risk of clinical uncertainty, misinterpretation, and missed opportunities for timely psychosocial or ethical intervention.

Addressing these shortcomings requires a coordinated, multi-level approach that builds on the perspectives of PPC professionals and aligns with existing research priorities in PPC that call for ethically informed responses to existential and communicative challenges. Conceptual clarity is needed to distinguish between normative reflections and expressions that signal psychosocial distress or an urgent need for support. Professional education should be expanded to cultivate both communicative and ethical competence, equipping teams to recognise and respond to DT and DW with confidence and sensitivity.

By strengthening institutional frameworks, improving professional preparedness, and investing in targeted research, PPC can more fully honour the voices of seriously ill children. In doing so, care becomes more consistent, ethically grounded and responsive to the emotional and moral complexity of supporting children and their families at the end of life.

## Supplementary Information

Supplementary Material 1.

Supplementary Material 2.

## Data Availability

The data sets used and/or analysed during the current study are available from the corresponding author on reasonable request, subject to compliance with data protection requirements.

## Abbreviations

PPC: Paediatric palliative care
HCP: Healthcare professionals
SOP: Standardised operating procedure
DT: Death thoughts
DW: Death wishes
DGP: Deutsche Gesellschaft für Palliativmedizin
