# Supplementary material for: Death wishes and death thoughts in paediatric palliative care: a survey of German healthcare professionals
Source: BMC Palliat Care. 2025 Dec 12;25:23. doi: 10.1186/s12904-025-01973-2 (PMC12817703; doi:10.1186/s12904-025-01973-2)
Supplement: Supplementary file 1 — Supplementary Material 1. [file 12904_2025_1973_MOESM1_ESM.docx]

# Supplementary Table 2.

# COREQ 32-Item Checklist (Adapted for Anonymous Online Survey Free-Text Data)

| Item | Guide question | Response |
| --- | --- | --- |
| 1. Interviewer/facilitator | Which authors conducted interviews or focus groups? | Not applicable – anonymous online survey; no interviews/focus groups. |
| 2. Credentials | Researcher credentials | FA (MD, PPC specialist, paediatric oncologist, clinical ethicist); MN (MD, researcher); HF (PhD, Public Health researcher); NWP (PhD, philosopher, researcher, senior clinical ethicist); CB (PhD, ethicist/supervisor); JF (MD, paediatric oncologist/supervisor) |
| 3. Occupation | Occupation at time of study | FA: paediatric oncologist/PPC specialist/ethicist; MN: physician/researcher; HF: Public Health researcher; NWP: university professor/clinical ethicist; CB: university professor of medical ethics; JF: university professor, senior clinician/academic in paediatric oncology |
| 4. Gender | Researcher gender | FA: female; MN: female; HF: female; NWP: male; CB: female; JF: male |
| 5. Experience/training | Experience in qualitative research | FA: PPC/ethics-trained, mixed-methods experience; MN: clinical & qualitative training; HF: formal qualitative methods training; NWP & CB: extensive qualitative ethics expertise; JF: clinical research supervision |
| 6. Relationship established | Was a relationship established beforehand? | Not applicable – anonymous survey |
| 7. Participant knowledge | What did participants know about the researchers? | Only study purpose provided; no personal researcher details due to anonymity |
| 8. Interviewer characteristics | Bias, assumptions, interests | Reflexive positions described in Methods section |
| 9. Methodological orientation | What underpinned the study? | Hybrid deductive–inductive thematic analysis. Deductive organisation used the predefined survey domains as an a priori organising framework, consistent with hybrid approaches in applied health research (Fereday & Muir-Cochrane, 2006; Nowell et al., 2017). Inductive, reflexive theme development followed Braun & Clarke’s principles of reflexive thematic analysis (2019). Methodological oversight was provided by senior qualitative researchers (NWP, CB) |
| 10. Sampling | How were participants selected? | Voluntary participation via PPC networks |
| 11. Method of approach | How were participants approached? | Survey link distributed via PPC mailing lists |
| 12. Sample size | Number of participants | 120 participants; subset provided qualitative free-text responses |
| 13. Non-participation | Refusals/dropouts | Not applicable – anonymous survey prevented tracking |
| 14. Setting | Where was data collected? | Online via LimeSurvey |
| 15. Presence of non-participants | Anyone else present? | Not applicable |
| 16. Description of sample | Important characteristics | Provided in Table 2 (roles, experience, demographics) |
| 17. Interview guide | Were questions provided/piloted? | Survey piloted; included open-text fields for qualitative data |
| 18. Repeat interviews | Repeat interviews? | Not applicable |
| 19. Recording | Audio/visual recording? | Not applicable |
| 20. Field notes | Field notes made? | Not applicable |
| 21. Duration | Interview duration? | Not applicable |
| 22. Data saturation | Was saturation discussed? | Not applicable for survey free-text data |
| 23. Transcripts returned | Transcript checking? | Not applicable |
| 24. Number of coders | How many data coders? | Two researchers (FA and MN) conducted the primary coding of all free-text responses. HF supported theme refinement, and NWP, CB, and JF provided methodological and supervisory oversight. |
| 25. Coding tree | Was a coding tree provided? | Not applicable – inductive semantic coding without hierarchical coding tree (consistent with reflexive TA) |
| 26. Derivation of themes | Were themes predefined or from data? | Themes were developed inductively from the free-text data after deductive organisation of responses into the predefined survey domains. Cross-domain refinement occurred through iterative reflexive team discussion. |
| 27. Software | Software used? | Manual coding; no software used |
| 28. Participant checking | Participants verify findings? | Not possible due to anonymity |
| 29. Quotations presented | Were quotes identified? | Yes , verbatim quotations are presented with anonymised identifiers (e.g., DT-02, DW-03). |
| 30. Consistency | Data and findings consistent? | Yes, each theme is supported by representative quotations illustrating the underlying analytic claims. |
| 31. Major themes | Are major themes clear? | Yes, three cross-cutting themes are presented clearly and systematically in the Results. |
| 32. Minor themes | Description of variation? | Yes, variations and nuances are described within each theme, appropriate to the scope and depth of a survey-based free-text dataset. |
