# Supplementary material for: Death wishes and death thoughts in paediatric palliative care: a survey of German healthcare professionals
Source: BMC Palliat Care. 2025 Dec 12;25:23. doi: 10.1186/s12904-025-01973-2 (PMC12817703; doi:10.1186/s12904-025-01973-2)
Supplement: Supplementary file 2 — Supplementary Material 2. [file 12904_2025_1973_MOESM2_ESM.docx]

# Supplementary Table 1.

**English translation of the Survey Instrument**

*The following table presents the English translation of the online survey instrument originally administered in German. The translation has been standardised for publication in BMC Palliative Care. It reflects the structure, wording, and logic of the original LimeSurvey questionnaire.*

| **Section** | **Item** | **Question** | **Response format** | **Open-text option** |
| --- | --- | --- | --- | --- |
| 1. Demographics | 1 | What is your professional background? | Single choice | No |
|  | 2 | How many years of experience do you have in paediatric palliative care? | Single choice | No |
|  | 3 | What is your gender? | Single choice | No |
|  | 4 | What is your age group? | Single choice | No |
|  | 5 | What is your religious affiliation? | Single choice | No |
| 2. Euthanasia inquiries | 6 | Have you ever been approached with questions about euthanasia in the context of paediatric palliative care? | Yes/No | No |
|  | 7 | If yes, how often? | Single choice (frequency scale) | No |
|  | 8 | Who initiated the inquiry? (patient, parents, siblings, etc.) | Multiple choice | No |
| 3. Assisted suicide | 9 | Have you ever received a direct request for assisted suicide from a paediatric patient? | Yes/No | No |
|  | 10 | If yes, how often? | Single choice (frequency scale) | No |
|  | 11 | What was the patient's age? | Single choice (age range) | No |
|  | 12 | What was the underlying diagnosis? | Single choice | No |
|  | 13 | Please recall or describe the wording of the request if you remember. | Open text | Yes |
| 4. Death thoughts (DT) | 14 | Have you encountered paediatric patients expressing thoughts about death? | Yes/No | No |
|  | 15 | How many patients have expressed such thoughts? | Single choice (frequency scale) | No |
|  | 16 | What was the age of these patients? | Multiple choice (age range) | No |
|  | 17 | Please recall or describe the wording of the child if you remember. | Open text | Yes |
| 5. Death wishes (DW) | 18 | Have you encountered paediatric patients expressing a wish to die? | Yes/No | No |
|  | 19 | How many patients have expressed such a wish? | Single choice (frequency scale) | No |
|  | 20 | What was the age of these patients? | Multiple choice (age range) | No |
|  | 21 | Please recall or describe the wording of the child if you remember. | Open text | Yes |
| 6. Communication practices | 22 | Do you actively address the topic of death and dying with paediatric patients? | Yes/No | No |
|  | 23 | Does your team have an SOP or internal guideline for conversations about death and dying? | Yes/No | No |
|  | 24 | Do you consider a clinical distinction between 'death thoughts' and 'death wishes' meaningful in paediatric palliative care? | Yes/No/Unsure | No |
|  | 25 | Please explain your reasoning. | Open text | Yes |
|  | 26 | What contributes to your uncertainty (if applicable)? | Open text | Yes |
| 8. Professional uncertainties (DT) | 27 | Do you think there is uncertainty among professionals in your interdisciplinary team when dealing with death thoughts? | Yes/No/Unsure | No |
|  | 28 | If yes, please describe the type of uncertainty observed. | Open text | Yes |
|  | 29 | What contributes to your uncertainty (if applicable)? | Open text | Yes |
| 9. Professional uncertainties (DW) | 30 | Do you think there is uncertainty among professionals in your interdisciplinary team when dealing with death wishes? | Yes/No/Unsure | No |
|  | 31 | If yes, please describe the type of uncertainty observed. | Open text | Yes |
|  | 32 | What contributes to your uncertainty (if applicable)? | Open text | Yes |
| 10. Ethical and institutional guidance | 33 | Do you believe that more research and institutional guidance on death-related expressions in paediatric palliative care are necessary? | Yes/No/Unsure | No |
|  | 34 | If yes, what kind of support or research would you recommend? | Open text | Yes |
|  | 35 | Can you explain your recommendation? | Open text | Yes |
|  | 36 | Please describe your reasoning. | Open text | Yes |
| 11. Recommendations (Outlook) | 37 | What specific measures or training would you recommend for improving professional responses to death-related expressions? | Open text | Yes |
|  | 38 | What ethical or institutional mechanisms could support professionals in this context? | Open text | Yes |
|  | 39 | What would you suggest to promote ethical reflection in PPC practice? | Open text | Yes |
|  | 40 | What topics should future research focus on in relation to DT and DW? | Open text | Yes |
|  | 41 | Would you support the development of structured ethical consultation pathways? | Yes/No | No |
|  | 42 | Would you participate in research or training addressing this topic? | Yes/No | No |
| 11. Recommendations (Outlook) | 43 | Any additional comments or recommendations? | Open text | Yes |
